# The protective effect and mechanism of catalpol on high glucose-induced podocyte injury

**DOI:** 10.1186/s12906-019-2656-8

**Published:** 2019-09-05

**Authors:** Yan Chen, Qingpu Liu, Zengfu Shan, Yingying Zhao, Meng Li, Baiyan Wang, Xiaoke Zheng, Weisheng Feng

**Affiliations:** 1 College of Pharmacy, Henan University of Chinese Medicine, Zhengzhou, Henan 450046 People’s Republic of China; 2 College of Basic Medicine, Henan University of Chinese Medicine, Zhengzhou, Henan 450046 People’s Republic of China; 3 Collaborative Innovation Center for Respiratory Disease Diagnosis and Treatment & Chinese Medicine Development of Henan Province, Zhengzhou, Henan 450046 People’s Republic of China

**Keywords:** *Rehmannia glutinosa*, Catalpol, Podocyte injury, Diabetic nephropathy, Mechanism

## Abstract

**Background:**

Catalpol, a natural iridoid glycoside in *Rehmannia glutinosa*, can alleviate proteinuria associated with diabetic nephropathy (DN), however, whether catalpol has a protective effect against podocyte injury in DN remains unclear.

**Methods:**

In this study, we used a high glucose (HG)-induced podocyte injury model to evaluate the protective effect and mechanism of catalpol against HG-induced podocyte injury. Cell viability was determined by the 3-(4,5-dimethylthiazolyl-2-yl)-2,5-diphenyltetrazolium bromide (MTT) method. The levels of lactate dehydrogenase (LDH), superoxide dismutase (SOD) and malondialdehyde (MDA) were measured by commercial assay kits. Cell apoptosis and reactive oxygen species (ROS) were determined by using flow cytometry. Tumour necrosis factor α (TNF-α), interleukin-1β (IL-1β) and interleukin-6 (IL-6) levels were determined by enzyme-linked immunosorbent assay (ELISA). The protein expression levels of B-cell lymphoma-2 (Bcl-2), Bcl2-associated x (Bax), cleaved caspase-3, nicotinamide adenine dinucleotide phosphate oxidase enzyme 4 (NOX4), toll-like receptor 4 (TLR4), myeloid differentiation primary response gene 88 (MyD88), p38 mitogen-activated protein kinase (p38 MAPK), phosphorylated p38 MAPK (p-p38 MAPK), nuclear factor kappa B inhibitor alpha (IκBα) and phosphorylated IκBα (p-IκBα) were measured by western blotting. In addition, Bcl-2, Bax, caspase-3 and nuclear factor kappa B (NF-κB) levels were determined by immunofluorescence staining.

**Results:**

Catalpol significantly increased cell viability and decreased LDH release in HG-induced podocyte injury. Catalpol significantly decreased ROS generation, apoptosis, level of MDA, levels of inflammatory cytokine TNF-α, IL-1β, and IL-6 and increased SOD activity in HG-induced podocyte injury. Moreover, catalpol significantly decreased expression of cleaved caspase-3, Bax, NOX4, TLR4, MyD88, p-p38 MAPK, p-IκBα and NF-κB nuclear translocation, as well as increased Bcl-2 expression in HG-induced podocyte injury.

**Conclusion:**

Catalpol can protect against podocyte injury by ameliorating apoptosis and inflammation. These protective effects may be attributed to the inhibition of NOX4, which alleviates ROS generation and suppression of the TLR4/MyD88 and p38 MAPK signaling pathways to prevent NF-κB activation. Therefore, catalpol could be a promising drug for the prevention of DN.

## Background

Diabetes mellitus is a common endocrine disease that leads to many dangerous complications, including diabetic nephropathy (DN). The number of DN patients is expected to rise to 642 million by 2040 [1, 2]. DN, one of the leading causes of end-stage renal disease (ESRD) worldwide, is characterized by the appearance of microalbuminuria as the earliest clinical manifestation [3, 4]. From a clinical perspective, although the treatments for hyperglycaemia and hypertension are the major treatments used for DN [5], these treatments are not enough to reverse the progression of DN.

Podocytes play a pivotal role in the pathogenesis of DN [6]. In its early stages, DN is primarily a glomerular disease, podocyte loss is generally found in DN patients and multiple studies have provided a correlation between podocyte loss and albuminuria in DN [7–9]. Podocytes, which are highly specialized and terminally differentiated visceral epithelial cells, make up the glomerular filtration barrier, cover the surface of the glomerular basement membrane, and play a key role in maintaining selective glomerular filtration [10]. Accumulating evidence suggests that podocyte injury is one of the main risk factors for DN, and that alleviating podocyte injury can improve DN [11–13]. However, there is a lack of effective therapeutic drug to alleviate podocyte injury.

**Fig. 1:**
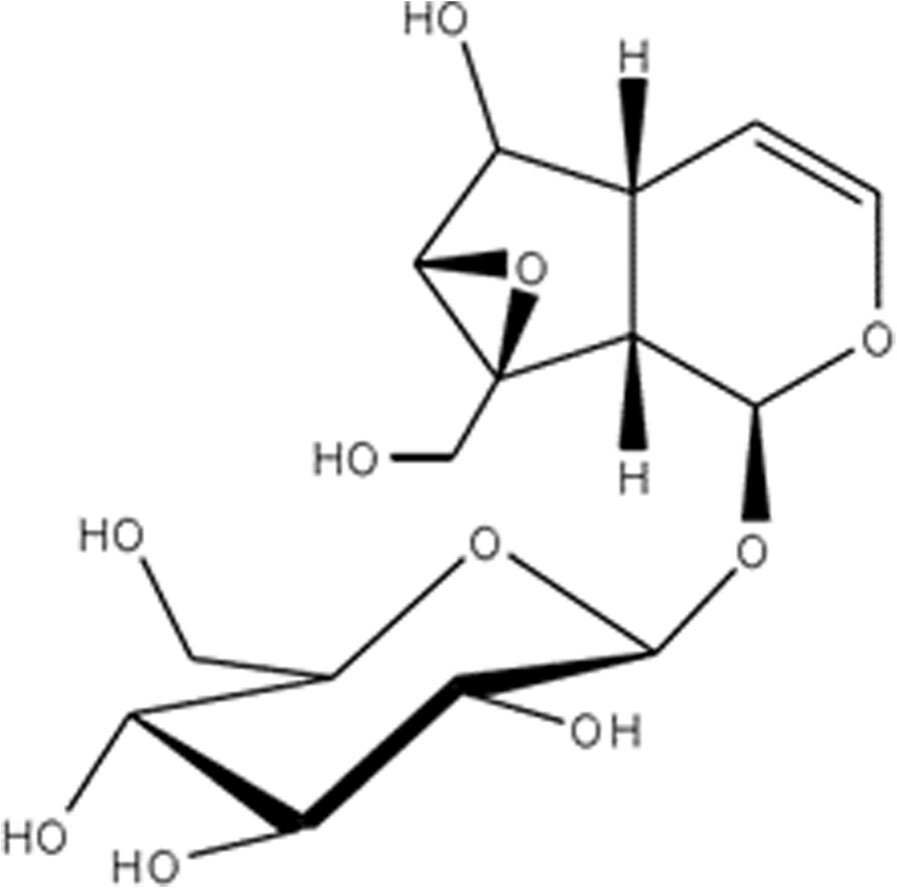
The structure of catalpol

Catalpol (Fig. 1), an iridoid glycoside, is the chief active component extracted from the root of *Rehmannia glutinosa*, which has long been used in traditional Chinese medicine. Catalpol has been reported to have a wide range of pharmacological activities, including those against DN [14, 15]. Several previous studies have shown that catalpol can improve renal function and decrease proteinuria, which is closely related to podocyte injury in DN [16, 17]. However, it is still unclear whether catalpol has a protective effect against podocyte injury in DN. In this study, we investigated the protective effect of catalpol in an HG-induced podocyte injury model and we further explored the possible mechanism of catalpol against HG-induced podocyte injury.

## Methods

### Materials

Catalpol was purchased from Nanjing Spring & Autumn Biological Engineering Co., Ltd. (Jiangsu, China), Roswell Park Memorial Institute (RPMI) 1640 medium was purchased from Gibco (Gibco Company, USA), and foetal bovine serum (FBS) was purchased from Scitecher Co., Ltd. (Oxford, MS, USA). Lactate dehydrogenase (LDH), malondialdehyde (MDA) and superoxide dismutase (SOD) kits were provided by Jiancheng Bioengineering Institute (Jiangsu, China). Glucose and reactive oxygen species (ROS) assay kit were purchased from Solarbio Life Sciences (Beijing, China). Antibodies for caspase-3 (ab13847), B-cell lymphoma-2 (Bcl-2) (ab59348), Bcl2-associated x (Bax) (ab32503), nicotinamide adenine dinucleotide phosphate oxidase enzyme 4 (NOX4) (ab133303), nuclear factor kappa B (NF-κB) (ab32536), TLR4 (ab22048) and MyD88 (ab135693) were purchased from Abcam (Cambridge, MA, USA). Antibody for β-actin (AC026) was purchased from Abclonal (Boston, USA). Antibodies for p38 mitogen-activated protein kinase (p38 MAPK) (#8690), phosphorylated p38 MAPK (p-p38 MAPK) (#4511), nuclear factor kappa B inhibitor alpha (IκBα) (#4814) and phosphorylated IκBα (p-IκBα) (#2859) were purchased from Cell Signaling Technology (Danvers, MA, USA).

### Cell culture

Conditionally immortalized mouse podocytes were provided by the National Infrastructure of Cell Line Resource (Beijing, China). Podocytes were cultured in RPMI 1640 medium supplemented with 10% FBS, 100 U/mL penicillin, 0.1 mg/ml streptomycin and 10 U/mL of mouse recombinant interferon-γ (IFN-γ) (PeproTech, California, USA) at 33 °C in a humidified atmosphere containing 5% CO_2_. Podocytes were cultured in RPMI 1640 medium without IFN-γ at 37 °C for 10–14 days to induce differentiation.

### Cell viability

Cell viability was determined by the 3-(4, 5-dimethylthiazolyl-2-yl)-2,5-diphenyltetrazolium bromide (MTT) method according to the manufacturer’s instruction. Briefly, podocytes were plated in 96-well plates at a density of 4 × 10^3^ cells/well and incubated with normal glucose (NG, 5.5 mM glucose+ 34.5 mM mannitol), high glucose (HG, 40 mM glucose) and HG with catalpol at different concentrations (1, 5, 10 μM) for 72 h. Mannitol (34.5 mM) was added to the NG group for osmotic control [18]. Then, an MTT solution (5 mg/mL) was added to each well, and the podocytes were incubated for 4 h at 37 °C. The supernatants were then aspirated, and 150 μL of dimethyl sulfoxide (DMSO) was added to solubilize formazan crystals with shaking for 10 min. Absorbance readings of the test and control samples were measured at 490 nm with a microplate reader (Thermo Scientific, Boston, USA) and cell viability is expressed in terms of the percentage viability, which was calculated as the ratio of the absorbance of a treated sample to the absorbance of the untreated control sample multiplied by 100.

### LDH assay

The release of LDH was measured by a commercial assay kit. Briefly, podocytes were plated in 96-well plates at a density of 4 × 10^3^ cells/well and incubated with NG, HG or HG with catalpol as described for the cell viability assay. At the end of the treatment, the culture medium was collected to assay LDH activity. Absorbance of test and control samples were measured at 450 nm with a microplate reader (Thermo Scientific, Boston, USA).

### Dichlorofluorescein assay to detect ROS

Intracellular ROS levels were measured using an ROS assay kit (Solarbio Life Sciences, Beijing, China*)*. Briefly, podocytes were seeded in 6-well plates at a density of 2× 10^5^ cells/well and incubated with NG, HG or HG with catalpol as described for the cell viability assay. Then, the podocytes were incubated with 10 μM 2′,7′-dichlorofluorescin diacetate (DCFH-DA) for 20 min at 37 °C. After the cells were washed three times with phosphate-buffered saline (PBS) solution to remove extracellular DCFH-DA, the fluorescence intensity was detected by flow cytometery (BD Biosciences, New York, USA).

### Annexin V/PI staining

Cell apoptosis was detected with FITC Annexin V apoptosis detection kits (BD Biosciences, New York, USA) according to the manufacturer’s instruction. Briefly, 1 × 10^6^ cells were harvested, washed, and resuspended in 500 μL of 1× loading buffer. Then, 5 μL of Annexin V-FITC and 5 μL of propidium iodide (PI) were added to the cells, which were incubated for 15 min in the dark at room temperature. Cell apoptosis was detected by flow cytometry (BD Biosciences, New York, USA).

### SOD and MDA assays

SOD and MDA were measured by using commercial assay kits according to the manufacturer’s instructions. In brief, podocytes were seeded in 6-well plates at a density of 2× 10 ^5^ cells/well and incubated with NG, HG or HG with catalpol as described for the cell viability assay. At the end of the treatment, the podocytes were washed with PBS three times and homogenized in 0.5 mL of buffer solution. The homogenates were centrifuged, and the supernatants were used to measure the SOD activity and MDA level. SOD activity and MDA level were detected in terms of absorbance readings measured at 450 nm and 530 nm wavelength respectively with a microplate reader (Thermo Scientific, Boston, USA).

### Determination of inflammatory cytokine levels in podocytes

Inflammatory cytokines, tumour necrosis factor α (TNF-α), interleukin-1β (IL-1β) and interleukin-6 (IL-6) levels in podocytes were determined using commercial ELISA kits (Biocalvin Science and Technology Ltd., Suzhou, China). All commercial kits were used according to the manufacturer’s instructions.

### Immunofluorescence staining

Bax, caspase-3, Bcl-2 and NF-κB levels were determined by immunofluorescence staining, which was performed according to methods described previously [19]. Briefly, after blocking with 5% BSA, podocytes were incubated with primary antibodies against Bax, caspase-3, Bcl-2 and NF-κB diluted 1:100. Alexa Fluor 488-conjugated goat anti-rabbit (Beyotime, Shanghai, China) or Alexa Fluor 555-conjugated goat anti-rabbit (Beyotime, Shanghai, China) were used as secondary antibodies. DNA was detected by staining for 5 min with DAPI (Beyotime, Shanghai, China), and the coverslips were mounted in glass shield (Beyotime, Shanghai, China). Caspase-3, Bcl-2 and Bax in podocytes were visualized by fluorescence microscopy (Nikon Corporation, Tokyo, Japan), and NF-κB in podocytes was visualized by confocal microscopy (Olympus Corporation, Tokyo, Japan).

### Western blot analysis

Western blotting was used to evaluate the levels of caspase-3, Bcl-2, Bax, NOX4, β-actin, TLR4, MyD88, p38 MAPK, p-p38 MAPK, IκBα, and p-IκBα protein. Briefly, podocytes were incubated with NG, HG or HG with catalpol as described for the cell viability assay. The podocytes were washed with PBS for three times and treated with 1 mL of radio immunoprecipitation assay (RIPA) lysis buffer containing phenylmethylsulfonyl fluoride. Supernatants were collected by centrifugation at 12,000 g for 30 min at 4 °C. The supernatants were mixed with loading buffer and boiled for 10 min. Then equal amounts of protein were loaded and separated by 10% sodium dodecyl sulfate-polyacrylamide gel, and the proteins were transferred from the gel onto polyvinylidene fluoride membranes. Then, the membranes were blocked with 5% nonfat dry milk in Tris-buffered saline containing 0.1% Tween-20 (TBST) for 1 h at room temperature. Primary antibodies were diluted in TBST (anti-Bax, 1:500; anti-cleaved caspase-3, 1:500; anti-Bcl-2, 1:500; anti-NOX4, 1:500; anti-TLR4, 1:1000; anti-MyD88, 1:1000; anti-p38 MAPK, 1:1000; anti-*IκBα*, 1:1000; anti-p-p38 MAPK, 1:1000; anti-p-IκBα, 1:1000; anti-β-actin, 1:5000) and incubated with the blots overnight at 4 °C. The blots were washed five times in TBST, treated for 1 h with IRDyeIgG (1:5000), and washed four times in TBST again. Densitometry analysis was performed as previously described [20].

### Data analysis

The results are expressed as the mean ± SD. Differences between two groups were assessed by one-way ANOVA followed by a post-hoc test (Fisher’s Least Significant Difference test) using SPSS 18.0 (IBM, New York, NY, USA). Differences were statistically significant when *p* < 0.05.

## Results

### Catalpol mitigates HG-induced podocyte injury

**Fig. 2:**
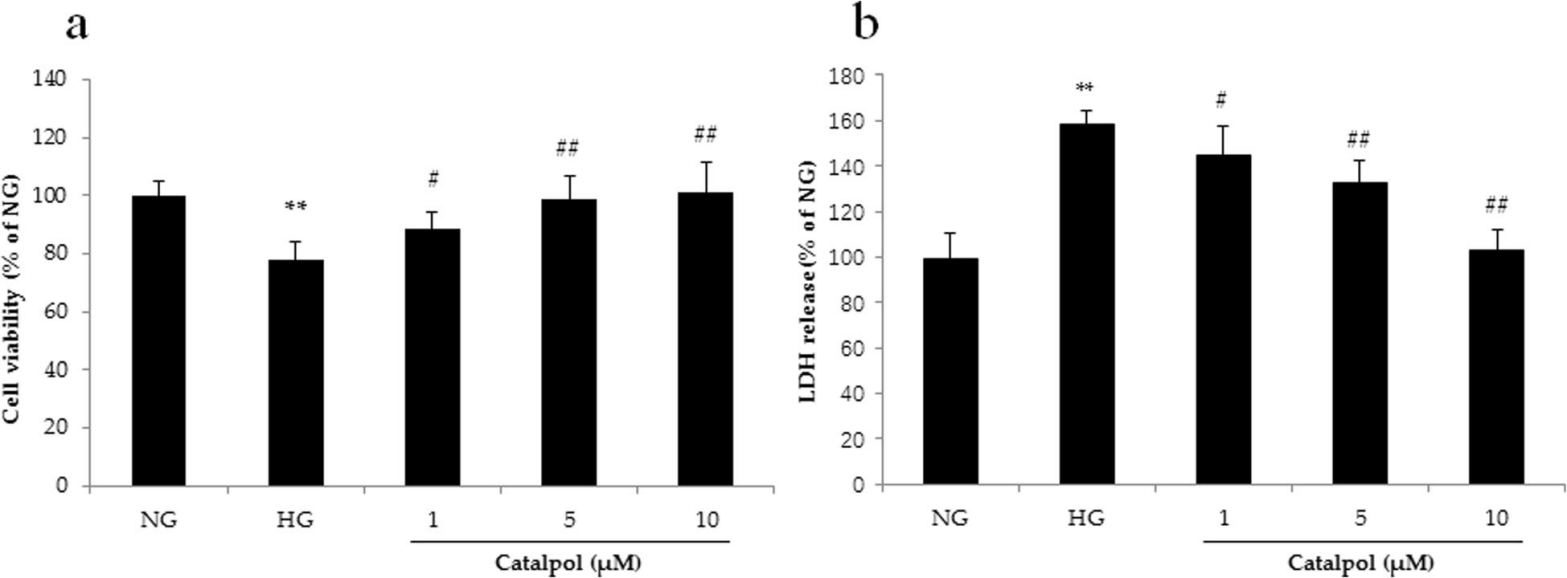
The protective effect of catalpol on HG-induced podocyte injury. **a** Cell viability (*n* = 6). **b** LDH release (*n* = 6). Data are presented as the mean ± SD. ** *p* < 0.01 vs NG, # *p* < 0.05, ## *p* < 0.01 vs HG

The treatment of podocytes with HG for 72 h induced cytotoxicity, as the cell viability decreased significantly compared to that of cells treated with normal glucose (NG) in the MTT test (Fig. 2a). When podocytes were treated with different concentrations (1, 5 and 10 μM) of catalpol, the cell viability was significantly increased. In the LDH release assay, HG significantly increased LDH release from podocytes, and catalpol (1, 5 and 10 μM) decreased LDH release from podocytes.

### The antioxidant effect of catalpol against HG-induced podocyte injury

**Fig. 3:**
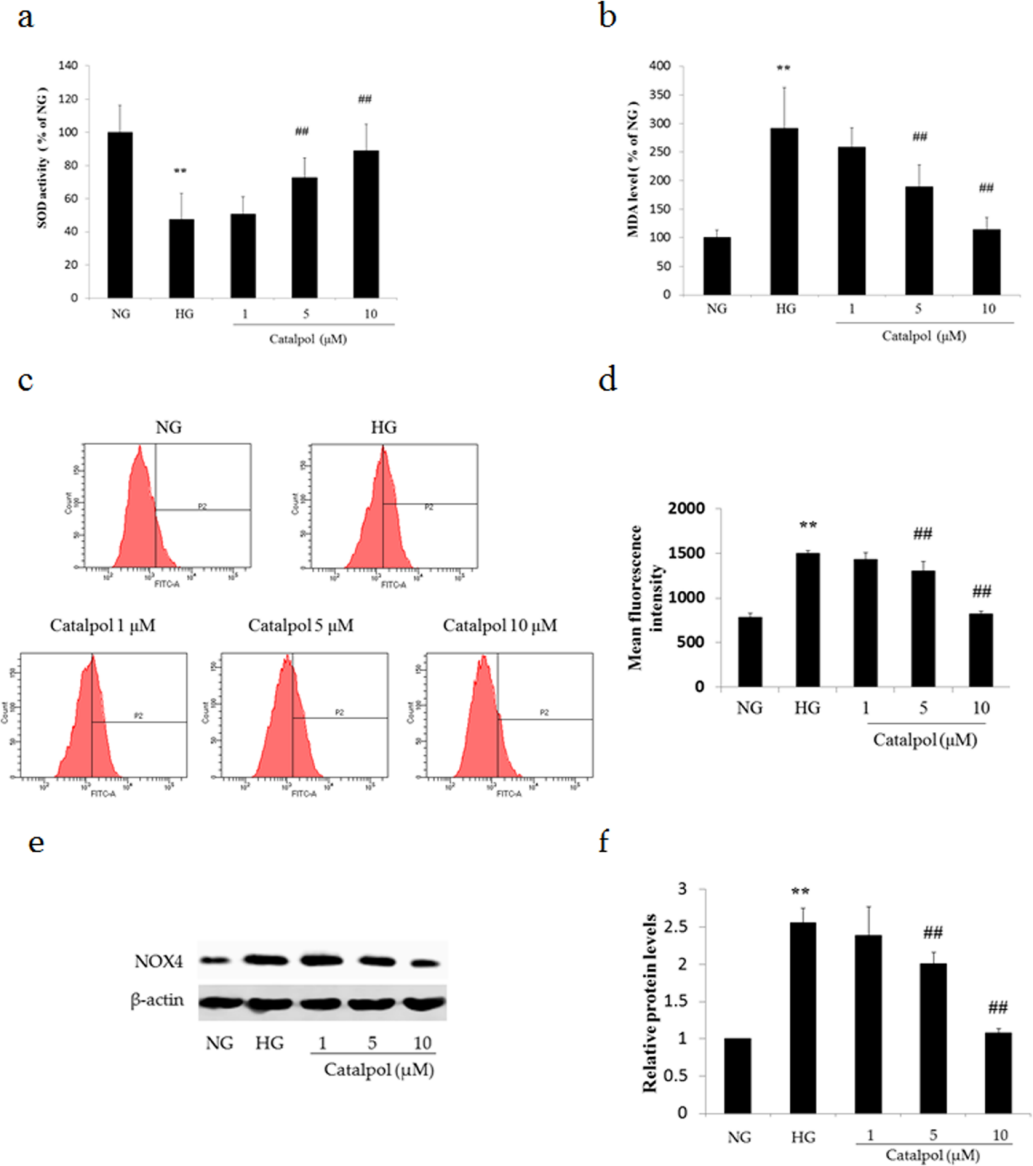
The antioxidant effect of catalpol on HG-induced podocyte injury. **a** The activity of SOD (*n* = 6). **b** The level of MDA (*n* = 6). **c** The ROS content of podocytes sorted by flow cytometry. **d** Histogram of the ROS content of podocytes (*n* = 3). **e** NOX4 detection in podocytes by western blotting. **f** Histogram of NOX4 expression relative to β-actin (*n* = 3). Data are presented as the mean ± SD. ** *p* < 0.01 vs NG, ## *p* < 0.01 vs HG

To determine the effect of catalpol on the antioxidant enzyme activity of podocytes under HG conditions, we assessed the SOD activity and MDA level. As shown in Fig. 3a,b, HG treatment significantly decreased SOD activity and increased MDA level in podocytes compared to those in podocytes treated with NG (*p* < 0.01). However, treatment with catalpol at 5 and 10 μM significantly increased SOD activity and decreased MDA level compared with those in the HG treatment group (*p* < 0.01). Moreover, we next assessed ROS generation in podocytes dyed with DCFH-DA (Fig. 3c, d). The ROS content of the HG treatment group was significantly increased compared to that in the NG treatment group (*p* < 0.01). However, catalpol suppressed ROS generation in podocytes exposed to HG (*p* < 0.01).

To assess the possible effect of HG on ROS-generating enzyme expression, we analysed the expression of NOX4, which is a key enzyme related to diabetes known to increase ROS levels in podocytes under various conditions [21]. As shown in Fig. 3e and f, HG treatment significantly elevated NOX4 expression compared with that following NG treatment, and catalpol (5 and 10 μM) treatment significantly reduced the expression of NOX4 in podocytes incubated with HG.

### The anti-apoptotic effect of catalpol against HG-induced podocyte injury

**Fig. 4:**
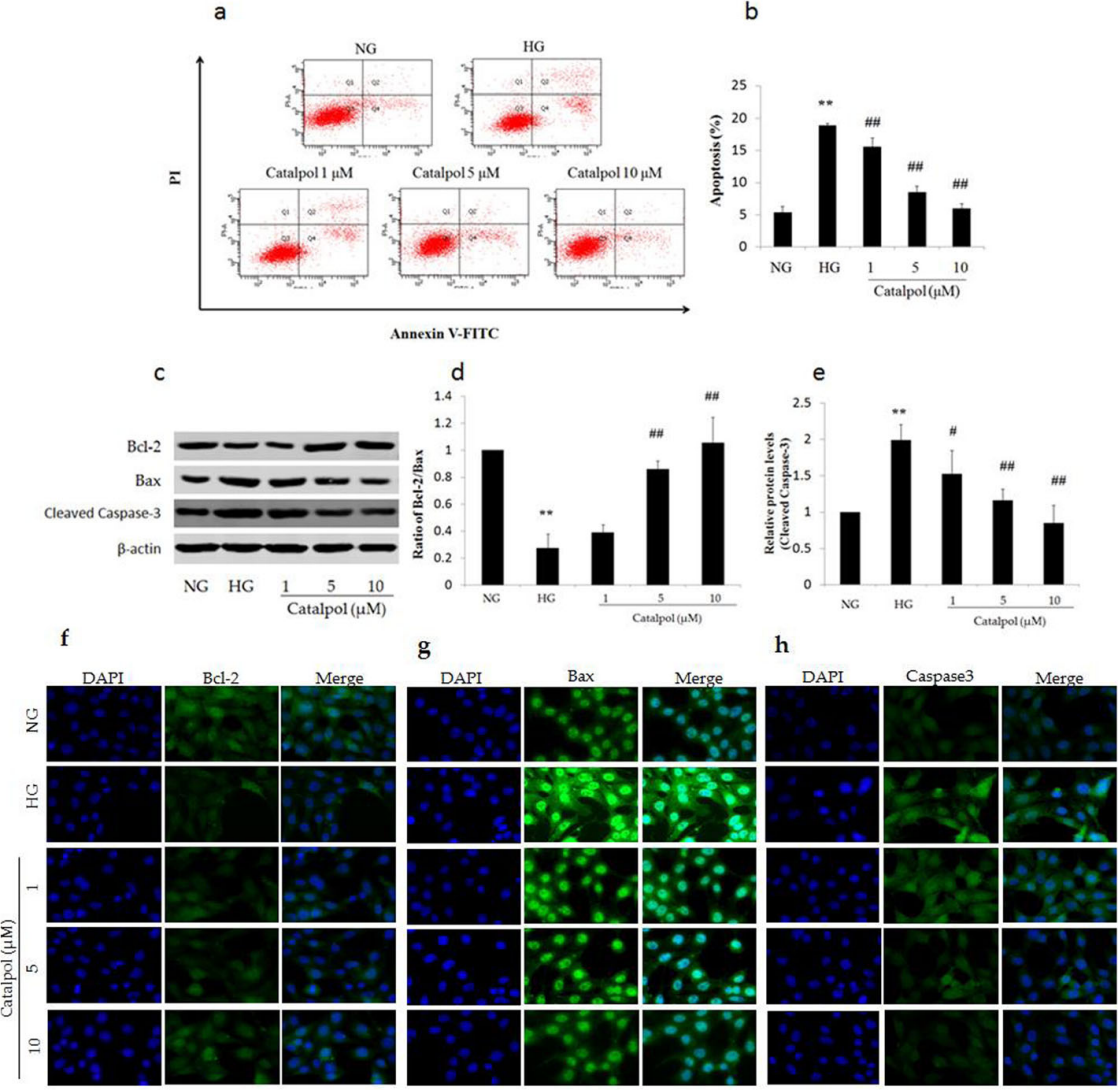
The anti-apoptotic effect of catalpol on HG-induced podocyte injury. **a** Populations of apoptotic podocytes sorted by flow cytometry. **b** Histogram of the apoptosis rate of podocytes (*n* = 3). **c** Bax, Bcl-2, and cleaved caspase-3 detection in podocytes by western blotting. **d** Histogram of the Bcl-2/Bax ratio in podocytes (*n* = 3). **e** Relative level of cleaved caspase-3 to β-actin in podocytes (*n* = 3). **f** Immunofluorescence staining for Bcl-2. **g** Immunofluorescence staining for Bax **h** Immunofluorescence staining for caspase-3. Data are presented as the mean ± SD. ** *p* < 0.01 vs NG, # *p* < 0.05, ## *p* < 0.01 vs HG

To elucidate whether the protective effect of catalpol is associated with reduced apoptosis, podocyte apoptosis was measured by flow cytometry. As shown in Fig. 4a, HG treatment significantly increased the percentage of apoptotic podocytes compared to that following NG treatment (*p* < 0.01), and catalpol treatment significantly decreased the extent of HG-induced apoptosis in podocytes (*p* < 0.01).

To further evaluate the anti-apoptotic role of catalpol in HG-induced podocyte injury, we measured Bcl-2, Bax and cleaved caspase-3 expression in podocytes using western blotting (Fig. 4c). As shown in Fig. 4d, HG treatment significantly decreased the ratio of Bcl-2 to Bax compared to that in the NG treatment group (*p* < 0.01), whereas catalpol treatment (5, 10 μM) significantly increased the Bcl-2/Bax ratio (*p* < 0.01) in podocytes exposed to HG. In addition, incubation with HG significantly increased cleaved caspase-3 expression in podocytes (*p* < 0.01), which was significantly attenuated by catalpol treatment (Fig. 4e). Moreover, immunofluorescence staining was performed to analyse the expression of Bcl-2 (Fig. 4f), Bax (Fig. 4g) and caspase-3 (Fig. 4h) in podocytes. The results of immunofluorescence staining were the same as those of western blotting, and showed that HG increased the expression of Bax and caspase-3 and decreased the expression of Bcl-2, while catalpol reversed these changes.

### The anti-inflammatory effect of catalpol in HG-induced podocyte injury

**Fig. 5:**
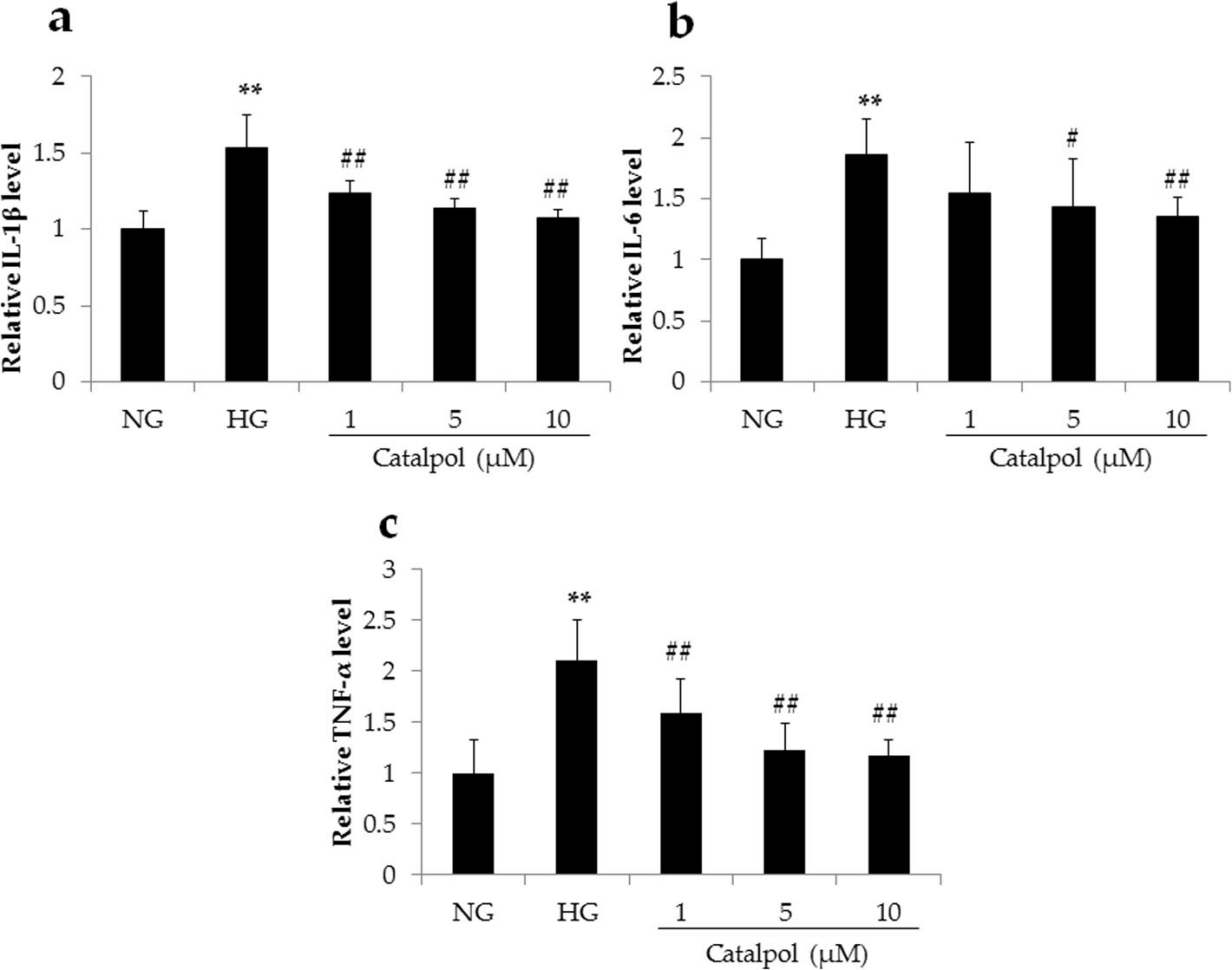
The anti-inflammatory effect of catalpol on HG-induced podocyte injury. **a** Relative level of IL-1β (*n* = 6). **b** Relative level of IL-6 (*n* = 6). **c** Relative level of TNF-α (*n* = 6). Data are presented as the mean ± SD. ** *p* < 0.01 vs NG, # *p* < 0.05, ## *p* < 0.01 vs HG

HG-induced podocyte injury is always associated with inflammation. To explore the anti-inflammatory effect of catalpol, we conducted ELISA to determine the IL-1β, IL-6 and TNF-α levels in podocytes exposed to HG, which were significantly higher in the HG-treated group than in the NG-treated group (*p* < 0.01), and catalpol treatment significantly decreased the levels of IL-1β, IL-6 and TNF-α in podocytes following HG treatment (Fig. 5).

### Effect of catalpol on TLR4/MyD88 signaling pathway in HG-induced podocyte injury

**Fig. 6:**
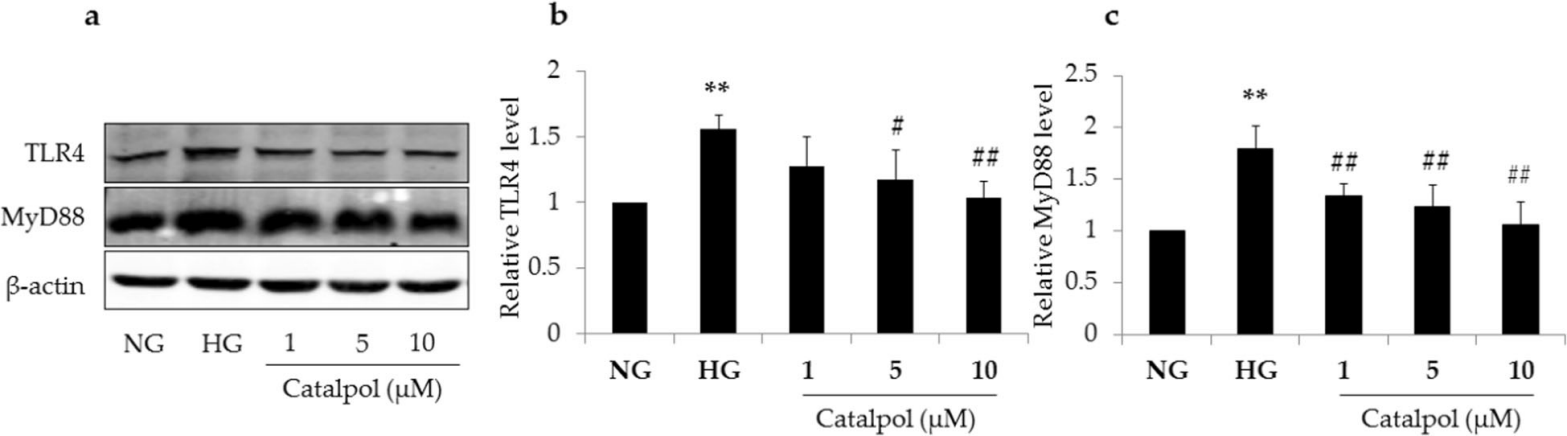
Effect of catalpol on TLR4/MyD88 signaling pathway in HG-induced podocyte injury. **a** TLR4 and MyD88 detections in podocytes by western blotting. **b** The relative level of TLR4 to β-actin in podocytes (*n* = 3). **c** The relative level of MyD88 to β-actin in podocytes (*n* = 3). Data are expressed as the mean ± SD. ** *p* < 0.01 vs NG, # *p* < 0.05, ## *p* < 0.01 vs HG

TLR4/MyD88 signaling pathway plays an important role in podocyte injury. In the present study, the expression of TLR4 and MyD88 in the HG group was significantly elevated compared with that in the NG group, as shown in Fig. 6 (*p* < 0.01), and catalpol treatment significantly decreased the expression of TLR4 and MyD88 in podocytes treated with HG. These results suggest that catalpol significantly inhibits the elevation of TLR4 and MyD88 in HG-induced podocyte injury.

### Effect of catalpol on ROS-mediated p38 MAPK/NF-κB signaling pathway in HG-induced podocyte injury

**Fig. 7:**
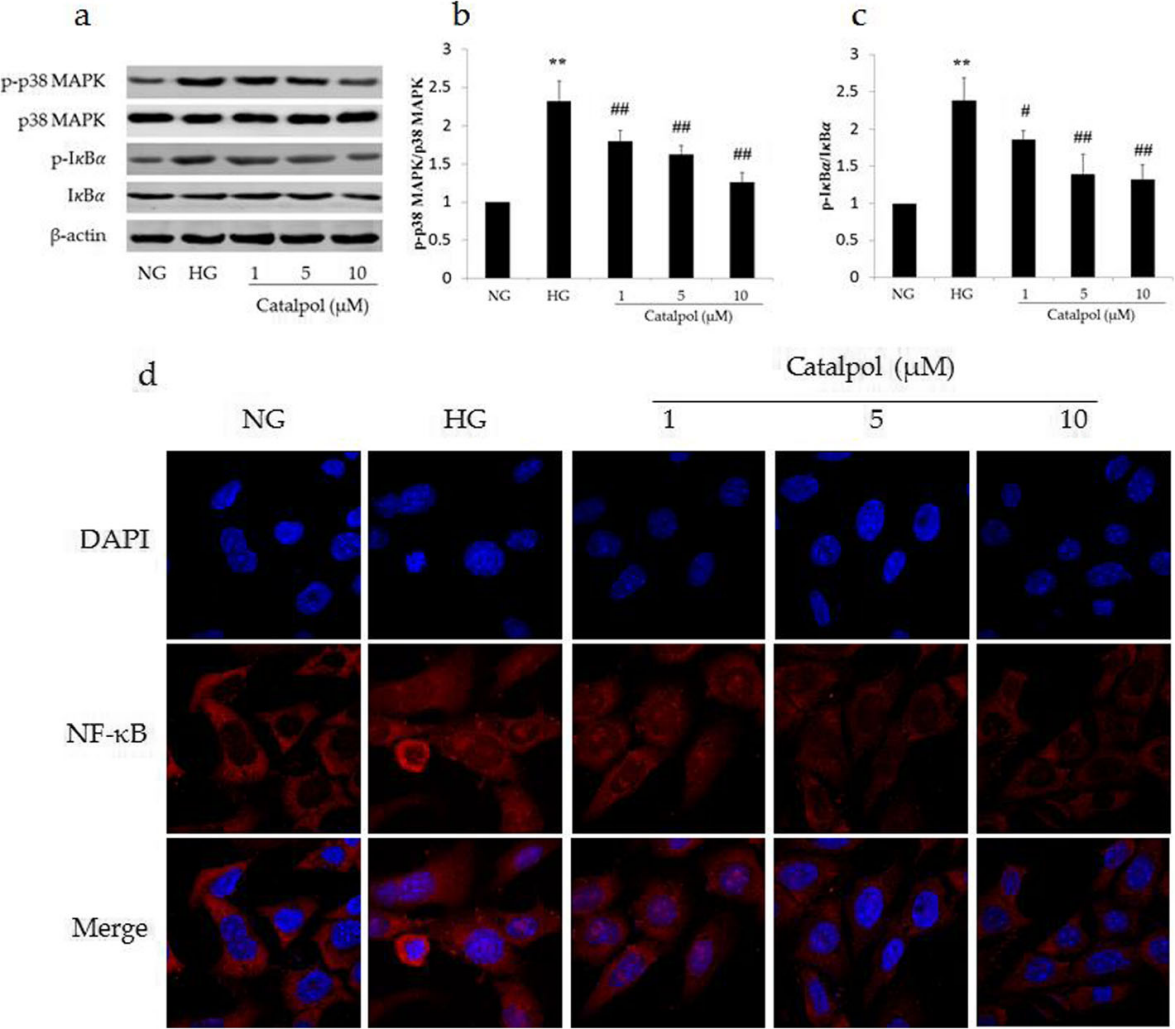
Effect of catalpol on the ROS-mediated p38 MAPK/NF-κB signaling pathway in HG-induced podocyte injury. **a** p38 MAPK, p-p38 MAPK, IκBα and p-IκBα detections in podocytes by western blotting. **b** Ratio of p-p38 MAPK/p38 MAPK in podocytes (*n* = 3). **c** Ratio of p-IκBα/IκBα in podocytes (*n* = 3). **d** Immunofluorescence staining for NF-κB. Data are presented as the mean ± SD. ** *p* < 0.01 vs NG, # *p* < 0.05, ## *p* < 0.01 vs HG

Since ROS can induce cell apoptosis via the p38 MAPK/NF-κB signaling pathway, we analysed the expression of p38 MAPK and IκBα and their phosphorylation. As shown in Fig. 7, although no significant change was observed in the expression of IκBα and p38 MAPK among all groups, treatment with HG significantly increased the levels of p-p38 MAPK and p-IκBα compared to those in the NG group, and catalpol treatment significantly inhibited the phosphorylation of p38 MAPK and IκBα compared with that in the HG group. In addition, immunofluorescence staining showed that HG significantly increased NF-κB translocation to nucleus and catalpol reversed this effect (Fig. 7d).

## Discussion

Catalpol has a wide range of pharmacological activities and has been demonstrated to exert protective effects against DN progression by ameliorating a variety of pathological changes. In the present study, the protective effect of catalpol on HG-induced podocyte injury was evaluated. Catalpol ameliorated apoptosis and inflammation by inhibiting NOX4 to alleviate ROS generation, and suppressing the TLR4/MyD88 and p38 MAPK signaling pathways to prevent NF-κB activation and translocation into the nucleus.

Podocytes are terminally differentiated cells, and podocyte injury is a pivotal event that leads to proteinuria in DN [22]. HG treatment dramatically decreased the cell viability and increased the percentage of apoptotic cells, which is consistent with the results of several studies [7, 23]. Furthermore, these damages were reversed by catalpol. Apoptosis, a tightly controlled process of cell death, is necessary for development and typical cell growth in organisms, and podocyte apoptosis is observed in vivo and in vitro models of DN [24, 25]. Bax and Bcl-2 are the key mediators of the intrinsic apoptotic response and cell apoptosis is stimulated by Bax and inhibited by Bcl-2 [26, 27]. Generally, the ratio of Bcl-2 to Bax is used to evaluate the degree of apoptosis; when this ratio decreases, apoptosis is stimulated. The anti-apoptotic effect of catalpol has been demonstrated in several models [28, 29]. In our study, HG treatment decreased the ratio of Bcl-2 to Bax in podocytes, resulting in their apoptosis, and catalpol increased the ratio of Bcl-2 to Bax in podocytes, showing that catalpol largely blocks apoptosis. In addition, several studies have demonstrated that the exposure of podocytes to HG can lead to inflammation [30, 31], which was confirmed in our study. HG treatment elevated the secretion of the pro-inflammatory cytokines IL-1β, TNF-α and IL-6, which was reversed by catalpol treatment. These results indicated that catalpol exerts a protective effect against HG-induced podocyte injury via its anti-apoptotic and anti-inflammatory effects.

Many stimulating factors, including ROS production, induce cell apoptosis [32]. ROS, the products of normal metabolism and xenobiotic exposure, are major factors associated with the pathogenesis of DN [33, 34] and crucial to the initiation of apoptosis in podocytes [35, 36]. Previous studies have confirmed that ROS are overproduced in podocytes in DN [37]. NOX4-dependent ROS production induces apoptosis in podocytes [38], which was observed in podocyte injury under DN conditions [39]. Choi et al. showed that catalpol could suppress the production of intracellular ROS elicited by AGE through inhibition of NADPH oxidase activity in human monocytic THP-1 cells [40]. In the present study, HG increased NOX4 expression and the intracellular ROS levels, which is consistent with the results of Fu et al. [41], and catalpol mitigated increased NOX4 and ROS. These results provide evidence that catalpol counteracts HG-induced ROS generation, indicating that the anti-apoptotic and anti-inflammatory effects of catalpol on HG-induced podocyte injury are associated with its antioxidant effect.

The p38 MAPK/NF-κB signaling pathway, which is regulated by ROS, plays an important role in apoptosis and inflammation [42–45]. The activation of p38 MAPK, a pro-apoptotic signaling factor downstream of ROS, leads to cell apoptosis [46]. Accumulating evidence suggests that excessive ROS can activate the pro-apoptotic p38 MAPK signaling pathway, which is associated with podocyte injury and proteinuria in DN [22]. In the present study, although there was no difference in the expression of p38 MAPK between NG-treated and HG-treated podocytes, the phosphorylation of p38 MAPK was significantly increased in HG-treated podocytes compared to that in NG-treated podocytes, and this phosphorylation was reversed by catalpol treatment. NF-κB is a crucial transcription factor involved in the progression of DN, and serves as an important downstream transcription factor of p38 MAPK activation [47, 48]. In the present study, p-I휅Bα and the nucleus translocation of NF-κB were increased significantly in HG-treated podocytes compared with those in NG-treated podocytes, however, both of these effects were inhibited by catalpol treatment. These results demonstrated that catalpol can suppress the p38 MAPK/NF-κB signaling pathway in HG-induced podocyte injury, which is related to its anti-apoptotic and anti-inflammatory effects of catalpol.

Moreover, TLR4/MyD88 signaling pathway plays an important role in inflammation in podocyte injury in vivo and in vitro models of DN [49–51], and TLR4 knockdown was shown to attenuate the increase in cell apoptosis in HG-induced podocyte injury [52]. TLR4 can recruit MyD88, which can also activate the NF-κB signaling pathway and cytokine production in podocyte injury [53, 54]. In the present study, the TLR4/MyD88 signaling pathway was activated, and cytokine production was observed in HG-induced podocyte injury, which is consistent with the results of several studies [52, 54]. Furthermore, catalpol significantly down regulated TLR4 and MyD88 expression and therefore prevented IκBα phosphorylation and degradation, possibly inhibiting NF-κB translocation into the nucleus and activation of the inflammatory response. These results indicated that catalpol can suppress TLR4/MyD88 signaling pathway activated in HG-induced podocyte injury to prevent NF-κB activation and translocation into nucleus and ultimately alleviate apoptosis and inflammation.

## Conclusions

This study demonstrated the anti-apoptotic and anti-inflammatory effects of catalpol on HG-induced podocyte injury. Those effects may be related to the inhibition of NOX4, which alleviates ROS generation, and suppression of the TLR4/MyD88 and p38 MAPK signaling pathways to prevent NF-κB activation and translocation into the nucleus. Therefore, catalpol could be a promising therapeutic agent for the treatment for podocyte injury against DN.

## Data Availability

The datasets used and analysed in the current study are available from the corresponding author upon reasonable request.

## Abbreviations

Bax: Bcl2-associated x
Bcl-2: B-cell lymphoma-2
DMSO: Dimethyl sulfoxide
DN: Diabetic nephropathy
ELISA: Enzyme-linked immunosorbent assay
ESRD: End-stage renal disease
FBS: Foetal bovine serum
HG: High glucose
IFN-γ: Interferon-γ
IL-1β: Interleukin-1β
IL-6: Interleukin-6
IκBα: Nuclear factor kappa B inhibitor alpha
LDH: Lactate dehydrogenase
MDA: Malondialdehyde
MTT: 3-(4, 5-dimethylthiazolyl-2-yl)-2,5-diphenyltetrazolium bromide
MyD88: Myeloid differentiation primary response gene 88
NF-κB: Nuclear factor kappa B
NG: Normal glucose
NOX4: Nicotinamide adenine dinucleotide phosphate oxidase enzyme 4
p38 MAPK: p38 mitogen-activated protein kinase
PI: Propidium iodide
p-IκBα: phosphorylated IκBα
p-p38 MAPK: phosphorylated p38 MAPK
RIPA: Radio immunoprecipitation assay
ROS: Reactive oxygen species
RPMI: Roswell Park Memorial Institute
SOD: Superoxide dismutase
TLR4: Toll-like receptor 4
TNF-α: Tumour necrosis factor α
